# Moxifloxacin versus Clindamycin/Ceftriaxone in the management of odontogenic maxillofacial infectious processes: A preliminary, intrahospital, controlled clinical trial

**DOI:** 10.4317/jced.52627

**Published:** 2015-12-01

**Authors:** Hansel Gómez-Arámbula, Antonio Hidalgo-Hurtado, Rosaura Rodríguez-Flores, Ana-María González-Amaro, Arturo Garrocho-Rangel, Amaury Pozos-Guillén

**Affiliations:** 1 DDS, Resident, Department of Orofacial Surgery; Hospital “Ignacio Morones Prieto”, San Luis Potosi, SLP, Mexico; 2 DDS, Associate Professor, Department of Orofacial Surgery; Hospital “Ignacio Morones Prieto”, San Luis Potosi, SLP, Mexico; 3 DDS, Associate Professor, Department of Oral and Maxillofacial Surgery, Faculty of Dentistry, San Luis Potosi University, San Luis Potosi, SLP, Mexico; 4 MS, Associate Professor, Biochemistry and Microbiology Laboratory, Faculty of Dentistry, San Luis Potosi University, San Luis Potosi, SLP, Mexico; 5 DDS, MS, PhD, Associate Professor, Basic Sciencies Laboratory, Faculty of Dentistry, San Luis Potosi University, San Luis Potosi, SLP, Mexico

## Abstract

**Background:**

The aim of this study was to compare the days of hospitalization length between patients treated with Moxifloxacin with that of patients treated with a Clindamycin/Ceftriaxone combination and additionally, to isolate and identify the oral pathogens involved in orofacial odontogenic infections.

**Material and Methods:**

A pilot-controlled-clinical-trial was carried out on hospitalized patients with cervicofacial odontogenic abscesses or cellulitis, who were randomly asigned to two study groups: 1) patients who received Moxifloxacin, and 2) patients receiving Clindamycin/Ceftriaxone combination. Infiltrate samples were collected through transdermic or transmucosal punction and later cultured on a media specific for aerobic and anaerobic microorganisms. Mean hospitalization duration in days until hospital discharge and susceptibility assessment in rates were established.

**Results:**

Mean hospitalization time in days of patients treated with Moxifloxacin was 7.0 ± 1.6 days, while in the Clindamycin/Ceftriaxone group, this was 8.4 ± 1.8 days, although significant difference could not be demonstrated (*p*=0.074). A total of 43 strains were isolated, all of these Gram-positive. These strains appeared to be highly sensitive to Moxifloxacin (97.5%) and Ceftriaxone (92.5%).

**Conclusions:**

Moxifloxacin and Ceftriaxone appear to be potential convenient and rational alternatives to traditional antibiotics, for treating severe odontogenic infections, in conjunction with surgical extraoral incision, debridement, and drainage.

** Key words:**Orofacial odontogenic infections, antimicrobial susceptibility, antimicrobial resistance.

## Introduction

Odontogenic infections are bacterial and inflammatory diseases that have affected human beings for centuries. They are the result of teeth with necrotic pulp tissue due to a periapical or periodontal lesion, pericoronal swelling, postsurgical infections, and direct trauma resulting in epithelial breach (1); they start as localized infections, but when these infectious processes are not well controlled, they can invade surrounding subcutaneous (s.c.) tissues, alveolar bone, and periostium via the periodontal margin and cause submucosal infiltrates and abscesses or cellulitis (2,3). These odontogenic infections can even penetrate into deep planes and aponeurotic spaces, which are ineffective anatomical barriers to the spread of infection, through the orofacial, intracraneal, mediastinal, and neck regions, producing serious, life-threatening septic complications, such as airway compromise, involvement of major blood vessels, bacterial endocarditis, pulmonary infections, cervical necrotizing fasciitis, necrotizing mediastinitis, up to retropharyngeal, orbital, and brain abscesses. Prognosis depends on the patient’s immunocompetence and the site of the inflammatory process (1,4). These are usually polymicrobial infections in nature, such as mixed aerobe/facultative anaerobe infections, and predominantly constituted by streptococci (5,6).

It has been established that management for odontogenic orofacial and neck infections is based on the surgical relief of purulence (6). However, antibiotics are an important adjunct during treatment of the infection, especially when the patient exhibits signs or symptoms of systemic involvement (3). Antimicrobial drug selection for odontogenic infections is mainly empirical and is based on its clinical effectiveness, minimal side effects, low costs, patient tolerability, and ready availability (3,7). Penicillin, Amoxicillin/clavulanic acid, and Clindamycin are some of the most widely used drugs, despite reports of allergic reactions, increasing resistance of microorganisms isolated from odontogenic abscesses or cellulitis, and other diverse systemic adverse reactions (8). In addition, Erythromycin, Tetracycline, and Levofloxacin have not proven very effective (7,9). Thus, other alternative antimicrobial agents for treating orofacial odontogenic infections are desirable.

Moxifloxacin is a tolerable, new, broad-spectrum 8-methoxy quinolone, with good oral-tissue penetration, bioavailability, and adequate resorption time after oral dosage, as demonstrated in animal models. It offers promising *in vitro* and clinical activity against odontogenic Gram-negative and multiresistant Gram-positive bacteria, aerobic and anaerobic bacteria, and atypical microorganisms, which is comparable to that of Amoxicillin/clavulanic acid (10-12). On the other hand, Ceftriaxone is a new, third-generation, semisynthetic cephalosporin with a long half-life that has resulted in the recommendation of the once-daily recommended, IV or intramuscularly (IM), administration schedule. It possesses a broad spectrum of activity against Gram-positive and -negative aerobic bacteria and some anaerobic bacteria. Ceftriaxone has been encouragingly effective in complicated and uncomplicated ear, nose, throat, skin, soft tissue, bone, and joint infections, with an apparent lack of serious side effects (13). In orofacial surgery, Ceftriaxone is often employed because it achieves high concentrations in bone and has shown to be an efficacious and cost-effective antimicrobial alternative to Penicillin G (IV) for mandible compound fracture antibiotic prophylaxis (14).

Considering the promising antimicrobial properties of Moxifloxacin and Ceftriaxone in orofacial surgery, with that Clindamycin still widely employed in the management of severe odontogenic infections, the aims of this preliminary clinical, controlled, and randomized trial were to: 1) compare the average duration in days of hospitalization length between patients treated with Moxifloxacin with those of patients treated with a Clindamycin/Ceftriaxone combination; 2) isolate and identify the oral pathogens involved in orofacial odontogenic infections; and 3) compare the efficacy and tolerability of Moxifloxacin with those of a Ceftriaxone/Clindamycin combination, administered as part of the management of orofacial odontogenic abscesses or cellulitis, in a Mexican hospitalized-patient sample.

## Material and Methods

-Patients

Hospitalized patients with severe orofacial odontogenic abscesses or cellulitis who required surgical and antimicrobial management were enrolled in the present study. Inclusion criteria were as follows: >18 years of age; good general status (American Society of Anesthesiologists [ASA] I or II); body temperature >38.5°C, pain (during swallowing or palpation); general involvement; trismus, and leukocytosis. Exclusion criteria were as follows: antimicrobial treatment prior to sample collection; pregnant or lactating patients; previous hypersensitivity or known adverse reactions to studied antibiotics; addiction to drugs, and immunosuppressive therapy (steroids, chemotherapy) within last 3 months.

-Study design

**Figure 1:**
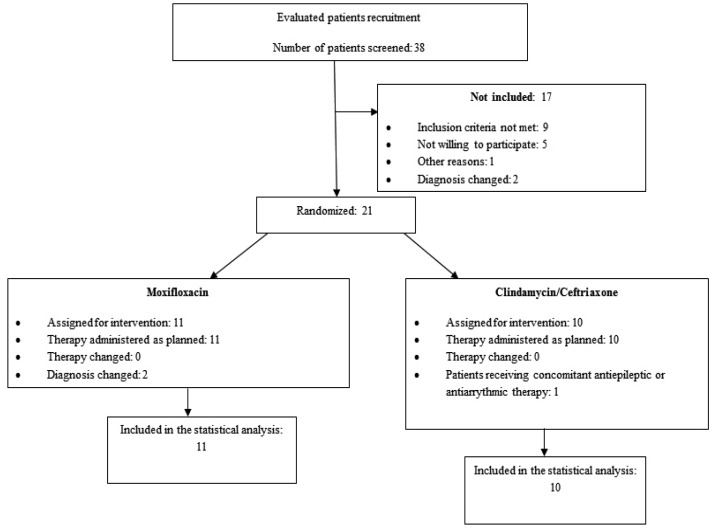
Description of study patient evolution according to Consolidated Standards of Reporting Trials (CONSORT) criteria.

This was a preliminary, monocentric, two-armed, controlled, unblinded clinical trial carried out following the guidelines suggested by the Consolidated Standards of Reporting Trials (CONSORT) group for planning and reporting clinical trials (Fig. 1) that was performed at the Orofacial Surgery Department of the Ignacio Morones Prieto Hospital in the city of San Luis Potosi, Mexico. The study was conducted in accordance with the Declaration of Helsinki and was approved by the hospital Ethics Committee. All of the subjects signed institutionally approved consent forms.

Initially and prior to beginning the treatment, baseline data, such as medical history and demographic information, were collected. Diagnostic tests and clinical and radiographic signs and symptoms (e.g., pain intensity, percussion, palpation, oral temperature, mouth opening, and the presence of lymphadenitis, swelling, erythema, or purulent secretion) were performed and assessed, in addition to drawing blood samples to measure basic laboratory parameters (blood biometry, blood chemistry, and blood clotting tests) (15). Thereafter, microbial sample collections and assigned treatments were conducted.

-Randomization

After obtaining the signed, informed-consent forms, the patients were randomly assigned to either study group for receiving the corresponding antimicrobial therapy, following the permuted-blocks randomization scheme: A, the Experimental group, and B, the Control group, in six different combinations of four-letter blocks. This method can ensure the approximate balance at end of recruitment, minimize the chance of imbalance due to unexpected shortfall in enrollment, and facilitate planning and implementation of the treatment administration process (16).

-Interventions

The antimicrobial therapy was as follows: experimental group, Moxifloxacin, 400 mg IV once daily until infection remission, and control group, Clindamycin 600 mg IV every 6 h plus Ceftriaxone 1 g IV two times daily, until infection remission. Before starting antimicrobial therapy and following careful skin disinfection with 1% povidone-iodine solution, an extraoral incision, tissue debridement, drainage placement, and microbiological sampling were performed. Concomitantly with the study drugs, patients could receive additional medical therapy, such as standardized analgesia, dental extractions, or supporting local procedures.

-Microbiological sampling and testing

Abscess samples were obtained through transdermal or transmucosal punction, employing a sterilized 10-mL hypodermic syringe, under a standard aseptic technique. Samples were immediately placed into anaerobe media, constituted of prereduced thioglycollate broth enriched with hemin and Menadione, and later incubated in an anaerobic atmosphere for 2-4 days. Then, all bacterial isolates were cultured on plates with anaerobic CDC agar added with 5% sheep blood for 3-5 days at a temperature of 37 ± 2°C, followed by their identification to the species level through colony characteristics, Gram staining, and employing the API 20 A, API 20 Strep, and API 20 Staph systems (bioMérieux, Durham, NC, USA). Antibiotic susceptibility and resistance testing of the isolates to assess antimicrobial agents was performed by the modified Kirby-Bauer method (17); microbial inhibition zones were measured and classified at three levels: resistant; intermediately sensitive, and highly sensitive, according to Clinical & Laboratory Standards Institute (CLSI) standards (18).

-Clinical efficacy assessment

Primary clinical outcome of this investigation was average duration in days, that is, from study entry until clinical remission of the infectious process (hospital discharge). This outcome was defined by the simultaneous occurrence of the following: body temperature <38.5°C; no palpatory pain; mouth opening similar or better than that at pretreatment, and normal white blood cell count. These criteria were evaluated daily. Mouth opening was measured as incisal edge distance in mm.

-Statistical analysis

Statistical analysis was initially performed at a descriptive level in terms of the demographic characteristics of the patient sample. After this, hypothesis tests were carried out to determine the differences between means in hospitalization-time length in days in both study groups, using the non-parametric Kruskal-Wallis test. Antimicrobial sensitivity was measured as rates, and the significance of the difference between rates was established through the goodness-of-fit Chi-square test. In both tests, the level of significance was established at 0.05.

## Results

A total of 21 patients were enrolled in the present clinical trial (52% female and 48% male), with an age rank of 20-69 years (mean age, 41.9 ± 8.3 years). At study initiation, 71.4% of patients exhibited a clinical abscess, while 28.6% were at cellulitis phase, with an evolution period with pain ranking between 2 and 15 days, and submandibular and buccal were the most affected aponeurotic spaces, in nearly 60% of cases. Odontogenic infections were more commonly caused by necrotic teeth localized in the mandible (53.8%) than in the maxilla (46.2%). Although two patients, one per study group, exhibited complications related with an infectious process (necrotizing fasciitis and mediastinitis with airway compromise, both mild) during the treatment period, all patients were successfully managed. No adverse events related with study antibiotics were reported in any study group.

Mean hospitalization time in the Moxifloxacin group was 7.0 ± 1.6 days, and in the control group, 8.4 ± 1.8 days, implicating no significant statistical difference between them (*p* = 0.074).

On the other hand, 43 bacterial strains were isolated in total, with a mean of 2.04 isolates per patient, with the predominance of facultative and moderate anaerobe microorganisms, such as streptococci (23.1%), aerococci (20.8%), and staphylococci (11.5%); Candida spp. was detected in one case. All isolated microorganisms were classified as Gram-positive ( Table 1).

**Table 1:**
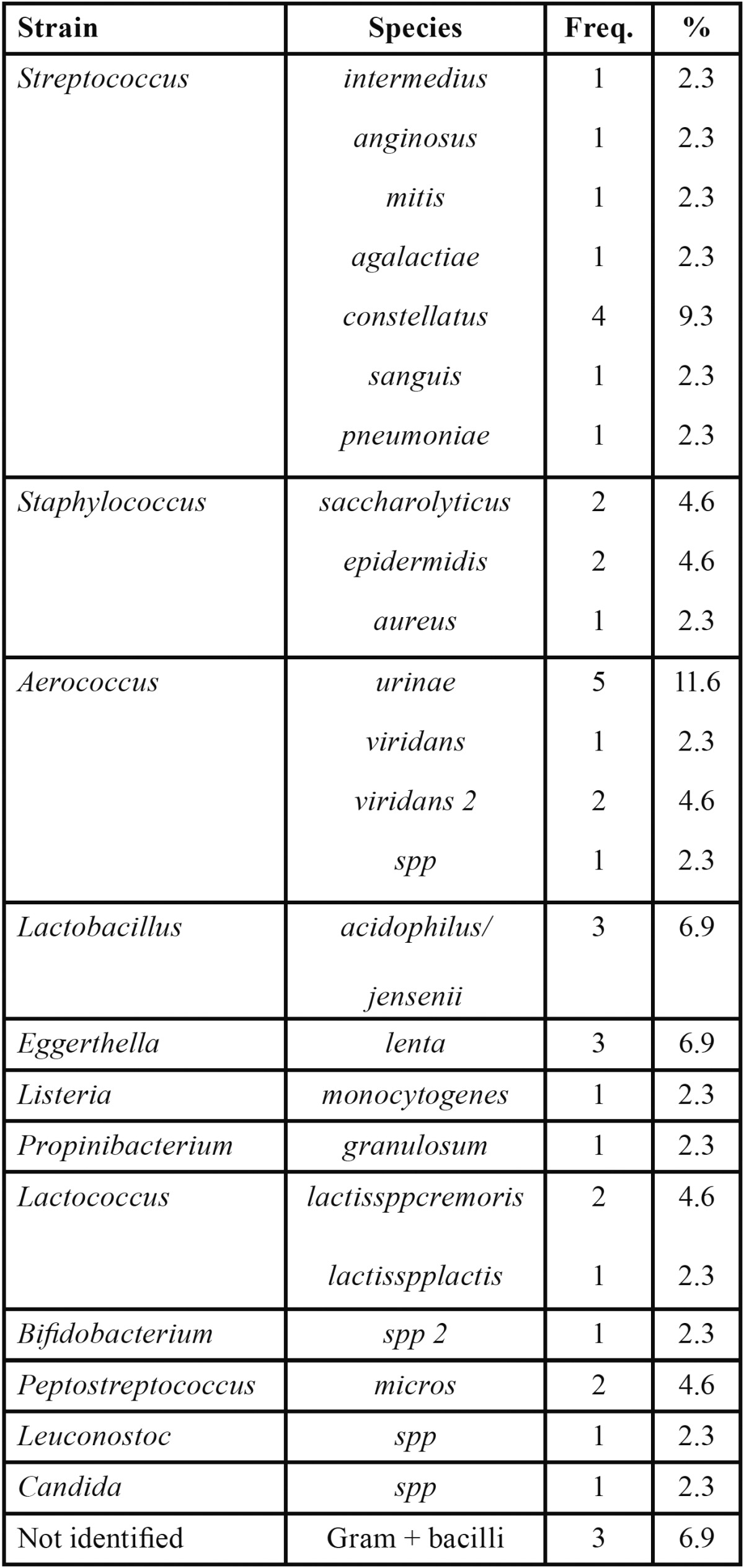
Frequency (Freq.) and rate of isolated and identified anaerobic microorganisms.

After obtaining a sufficient microbial growth, susceptibility tests showed that Moxifloxacin and Ceftriaxone exhibited significantly better results than Clindamycin ( Table 2).

**Table 2:**
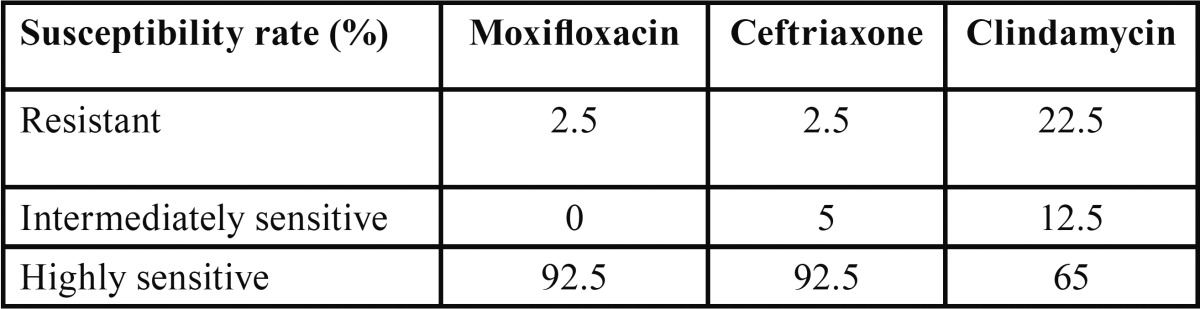
Microbial susceptibility rates. Isolated bacteria exhibited significantly higher susceptibility (and lower resistance) to Moxifloxacin and Ceftriaxone with respect to Clindamycin (*p*<0.01). Between these two antibiotics, there was not a significant diference (*p* = 0.08).

## Discussion

In recent years, diverse new antibiotics have been developed, but none have demonstrated better efficacy or benefit in order to replace the use of Penicillin for the treatment of these infectious processes. The judicious decision to employ a specific antibiotic agent when managing a severe odontogenic infection should be based on several factors; undoubtedly, the precise clinical diagnosis and the etiology are the most important of these factors (1,8). In addition, the clinician must collect information concerning host-defense mechanisms, infection severity and extension, and expected casual pathogens (7). The microbial susceptibility data are also crucial, but it may take several days for these to be obtained; thus, a pragmatic, rational approach to empirical antibiotic selection is currently acceptable, provided the antibiotic choice is based on scientific data and contemporary experience in managing orofacial infections (19).

According to the results generated in the present study, there were three main findings: a) in all cases, both antibiotic schemes were equally effective in a relatively short time for resolution of odontogenic infections; b) moderate anaerobic and facultative anaerobic flora was mainly found in the microbiologic samples from these odontogenic infections, and, c) the microorganisms isolated were significantly less resistant to Moxifloxacin and Ceftriaxone with respect to Clindamycin. In addition, we should mention that all study antibiotics were well tolerated, considering that there was no any adverse effect related with them.

According to Swift *et al.* (7) the average time-period length for odontogenic infection treatment until remission is between 5 and 7 days, similar to that in the present study; another study was carried out in a Finnish sample of 35 patients under hospital care, with a similar mean age to ours, of which 25 exhibited local abscesses and cellulitis formations without systemic complications. Mean length of hospital stay was 8.0 days (range, 2-34 days), equivalent to the value observed here (20). In addition to being cost-effective, especially for hospitalized patients, the relatively short therapy-time length with Moxifloxacin and Ceftriaxone decreases the risk of microbial development of resistance and enables patients to return to work earlier. Moxifloxacin and Ceftriaxone possess the advantage of being administered only once daily, thus enhancing patient compliance, unlike other antibiotic schemes that comprise several takes during the day; additionally, the lower daily doses of Moxifloxacin (400 mg) and ceftriaxone (1 g), in comparison with those of Clindamycin (2,400 mg) reduce the metabolic load in the patients affected (12).

Odontogenic infection is the product of the interdependent and synergistic metabolism of the diverse pathogenic microorganisms involved: individual members within a group generate metabolites that are essential for an appropriate growth environment of other microorganisms in the group, including favorable pH and available nutrients and oxygen levels (1,7,8). Our observations are in agreement with the findings of previous studies, in which the number of microbial isolates per patient ranged from 2-5 (9). Those studies also reported a polymicrobial constituted of anaerobic and facultative anaerobic bacterial flora; however, our samples nearly exclusively comprised Gram-positive facultative anaerobic microorganisms, mainly streptococci, aerococci, and staphylococci, and including only a few anaerobic species, which could explain the excellent antimicrobial activity exhibited by Moxifloxacin and Ceftriaxone (5); our findings are similar to those reported by Singh *et al.* (1) According to Walia *et al.* (3) staphylococci are frequently associated with odontogenic abscess formation and typically produce the enzyme coagulase, which cause fibrin deposition in citrated or oxalated blood; on the other hand, streptococci also produce enzymes such as streptokinase (fibrinolysin), hylouronidaze, and streptodornase, breaking down fibrin, connective-tissue ground substance, and lysing cellular debris. All of these facts render the rapid spread of infectious microbes into deeper planes (3,8).

Moxifloxacin, but especially Ceftriaxone, have been scarcely studied in cases of severe odontogenic infections, in which facultative anaerobic and anaerobic microorganisms are thought to play a central role. Moxifloxacin antimicrobial activity depends on the inhibition of DNA gyrase and on its well-documented good penetration into spongy and compact bone and muscle tissues; it has shown a minimal inhibitory concentration required to inhibit the growth of 90% of organisms (MIC90) of between 0.064 to 0.5 mg/l against Viridans streptococci, (2,11) achieving good odontogenic-site concentrations and exceeding the plasma concentrations (2,4). On the other hand, Ceftriaxone possesses bactericidal activity against group A and group B streptococci and peptostreptococcus, and slightly lower action than that of Penicillin against Gram-positive cocci, all of these pathogen components isolated from odontogenic infectious processes; this antimicrobial agent acts by inhibiting cell-wall synthesis and possesses a MIC90 of 0.125 mg/l against isolated streptococci (11,14). As demonstrated here and in previous studies, and due to their adequate pharmacokinetic properties, both antimicrobial agents exhibit excellent tolerability, long half-life, and high bioavailability for their employment in the treatment of odontogenic cervicofacial infections (2,4).

Our results concerning Moxifloxacin and Clindamycin resistance are according to those of Warnke *et al.* (21) and Sobottka *et al.* (4) with nearly 100% of all anaerobe/facultative anaerobe isolates susceptible to Moxifloxacin, whereas 65% of these were susceptible to Clindamicyn. Compared with Penicillin, Tomás *et al.* (11) reported, from a study in which bacteria were isolated from blood following dental extractions, that >10% of the streptococci were resistant to this antibiotic, whereas >20% were resistant to Clyndamycin, and no bacteria isolates were resistant to Moxifloxacin. All of these data demonstrate the very high susceptibility to Moxifloxacin of pathogen microorganisms isolated from severe orofacial, odontogenic infectious processes.

Moxifloxacin and Ceftriaxone appear to be potential convenient and rational alternatives to traditional antibiotics, for treating severe odontogenic infections, in conjunction with surgical extraoral incision, debridement, and drainage. New studies should consider a larger number of patients, in order to evaluate if the observed trend is significant.

In conclusion, there were three main findings: a) in all cases, both antibiotic schemes were equally effective in a relatively short time for resolution of odontogenic infections; b) moderate anaerobic and facultative anaerobic flora was mainly found in the microbiologic samples from these odontogenic infections, and, c) the microorganisms isolated were significantly less resistant to Moxifloxacin and Ceftriaxone with respect to Clindamycin.
